# Conversion disorder in children and adolescents : Clinical features of pseudoneurological symptoms

**DOI:** 10.1192/j.eurpsy.2021.490

**Published:** 2021-08-13

**Authors:** R. Gadhoum, M. Daoud, H. Ben Rhouma, S. Ben Salem, H. Klaa, I. Kraoua, I. Turki

**Affiliations:** 1 Department Of Neuropediatrics, Neurology National Institute, Tunis, beeb saadoun, Tunisia; 2 Department Of Neuropediatrics, Neurology National Institute, Tunis, Beb Sadoun, Tunisia; 3 Psychiatry G, Hospital of psychiatry, manouba, Tunisia

**Keywords:** Psychogenic, conversion, pseudoneurological, Children

## Abstract

**Introduction:**

Pseudoneurological symptoms are frequent among children consulting in neuropediatrics. Psychogenic origin is often unrecognized, which may cause a major disruption and an increase of medical care expenses.

**Objectives:**

The purpose of this study was to identify clinical features of pseudoneurological symptoms through patients admitted in neuropediatrics.

**Methods:**

A descriptive retrospective study of a population of 19 children and adolescents hospitalized in the neuropediatrics department at the National Institute of Neurology in Tunis, between January 2015 and April 2019, having recieved the diagnosis of psychogenic symptoms.

**Results:**

Twelve girls and seven boys were included in this study.The averge age were 11.5 years. All patients had normal cogntive and motor development. In most cases (84%), patients had a history of somatic illness. Only three patients had a history of psychiatric disorders. Family history of somatic disorders was found in 42 % of the sample and psychiatric disorders in three patients. Negative pseudoneurological symptoms such as loss of function, were detected in 60 % of patients, paraparesis and paraplegia were the most recurrent. Only one patient had pseudo-epileptic symptoms. Further investigations were performed in all patients, averaging 4 tests per patient. The average term between the beginning of the symptoms and the established diagnosis of psychogenic symptoms was 72 days with an average stay at hospital of 4 to 7 days. All patients had conversion disorder according to DSM V.

**Conclusions:**

It is recognized that somatization could be a warning sign of psychological distress mainly among children. Conversion disorder, rarely seen in children, presents frequently as pseudo neurological symptoms.

